# Supplementary material for: OTX2 Duplication Is Implicated in Hemifacial Microsomia
Source: PLoS One. 2014 May 9;9(5):e96788. doi: 10.1371/journal.pone.0096788 (PMC4016008; doi:10.1371/journal.pone.0096788)
Supplement: Table S1 — Array and sequencing concordance. The probabilities to observe genetic variants in the sequencing data conditioned on the array data status and collapsed in all three individuals. (DOCX) [file pone.0096788.s005.docx]

**Table S1**

|  | | **Sequencing data** | |
| --- | --- | --- | --- |
|  |  | **Ref** | **Non-ref** |
| **Array data** | **Ref** | 0.99719561 | 0.00280439 |
|  | **Non-ref** | 0.01714976 | 0.98285024 |
