# Supplementary material for: OTX2 Duplication Is Implicated in Hemifacial Microsomia
Source: PLoS One. 2014 May 9;9(5):e96788. doi: 10.1371/journal.pone.0096788 (PMC4016008; doi:10.1371/journal.pone.0096788)
Supplement: Table S2 — Exome sequencing quality metrics. A summary of the quality control indicators from the three exome sequencing datasets. (DOCX) [file pone.0096788.s006.docx]

**Table S2**

|  | **III.1** | **V.2** | **V.3** |
| --- | --- | --- | --- |
| **# lanes (Illumina)** | 2 | 3 | 3 |
| **Average coverage** | 95.32x | 99.24x | 105.26x |
| **Exome covered** | 96.00% | 97.30% | 96.90% |
| **Exome covered ≥ 5x** | 90.40% | 91.60% | 91.30% |
| **Ts/Tv rate** | 3 | 2.89 | 2.93 |
| **Homozygous:heterozygous** | 0.57 | 0.58 | 0.56 |
