# Supplementary material for: OTX2 Duplication Is Implicated in Hemifacial Microsomia
Source: PLoS One. 2014 May 9;9(5):e96788. doi: 10.1371/journal.pone.0096788 (PMC4016008; doi:10.1371/journal.pone.0096788)
Supplement: Table S3 — Candidate variants. We retained only variations shared IBD in individuals III.1, V.2, and V.3. (DOCX) [file pone.0096788.s007.docx]

**Table S3**

| **chr** | **pos** | **observed alleles** |
| --- | --- | --- |
| 1 | 1334409 | C/G |
| 1 | 1900106 | insCCT |
| 1 | 229462617 | G/T |
| 2 | 129075877 | G/T |
| 2 | 130832185 | A/A |
| 2 | 130832292 | A/T |
| 3 | 12983199 | A/G |
| 3 | 56650051 | insCTT |
| 8 | 6673377 | -A |
| 8 | 6679498 | G/T |
| 8 | 7308386 | C/C |
| 8 | 7673126 | A/C |
| 8 | 8887542 | delAAC |
| 8 | 10467652 | C/G |
| 8 | 11995570 | G/T |
| 8 | 12878927 | A/T |
| 8 | 64098729 | insG |
| 8 | 86126827 | insAACATT |
| 9 | 894197 | G/T |
| 9 | 21077767 | C/G |
| 10 | 97920099 | insC |
| 10 | 118383463 | insG |
| 10 | 126683123 | A/C |
| 10 | 126683151 | C/T |
| 12 | 7080210 | insG |
| 12 | 7456988 | C/T |
| 12 | 8327883 | C/T |
| 12 | 8374781 | -/ACG |
| 12 | 9994445 | delTGT |
| 12 | 10332200 | A/G |
| 12 | 10573094 | C/G |
| 12 | 10588530 | C/G |
| 12 | 11149585 | A/C |
| 12 | 11244149 | A/G |
| 12 | 11420333 | -/G |
| 12 | 11506669 | G/T |
| 12 | 18435398 | -/CCC |
| 12 | 55523586 | -T |
| 12 | 57433048 | C/T |
| 12 | 75816814 | insACA |
| 15 | 22074657 | C/G |
