# Supplementary material for: OTX2 Duplication Is Implicated in Hemifacial Microsomia
Source: PLoS One. 2014 May 9;9(5):e96788. doi: 10.1371/journal.pone.0096788 (PMC4016008; doi:10.1371/journal.pone.0096788)
Supplement: Table S4 — Sanger sequencing results of the four genes with biological activity that could be attributed to HFM. (DOCX) [file pone.0096788.s008.docx]

**Table S4**

| **gene** | **chr** | **loc** | **ref** | **alt** | **V.2** | **V.3** | **III.1** | **III.3** |
| --- | --- | --- | --- | --- | --- | --- | --- | --- |
| **DAB2** | 5 | p13.1 | T | C | C/T | C/T | C/T | T |
| **IQSEC1** | 3 | p25.2 | G | A | A/G | A/G | A/G | G |
| **KIAA1456** | 8 | p22 | T | A | A/T | A/T | A/T | T |
| **ADAM28** | 8 | p21.2 | T | C | C/T | C/T | C/T | T |
